# The Psychometric Properties of the Braden Scale to Assess Pressure Injury Risk in Acute Care: A Systematic Review

**DOI:** 10.1111/jocn.17862

**Published:** 2025-06-16

**Authors:** Adam Burston, Jacob Butterworth, Aldiana Mehicic, Paul Fulbrook

**Affiliations:** ^1^ Nursing Research and Practice Development Centre The Prince Charles Hospital Chermside Queensland Australia; ^2^ School of Nursing, Midwifery and Paramedicine (Brisbane), Faculty of Health Sciences Australian Catholic University Brisbane Queensland Australia; ^3^ School of Therapeutic Sciences, Faculty of Health Sciences University of the Witwatersrand Johannesburg South Africa

**Keywords:** acute care, Braden scale, pressure injury, pressure ulcer, risk assessment, systematic review

## Abstract

**Aim:**

To analyse existing knowledge on the psychometric properties of the Braden Scale when used within the acute care setting.

**Design:**

Systematic review and narrative synthesis.

**Methods:**

A database search was conducted in June 2023 and updated in February 2024, seeking studies testing the psychometric properties of the Braden scale in the acute care setting. Data were sourced from five electronic databases (CINAHL, EMBASE, MEDLINE, Scopus and Web of Science). Study selection, data extraction and assessment of risk of bias were completed, with two reviewers independently conducting each stage and an independent reviewer arbitrating discrepancies. Data were extracted using a customised template and synthesised narratively. Risk of bias was assessed using the COnsensus‐based Standards for the selection of health Measurement INstruments (COSMIN) checklist.

**Results:**

Thirty‐seven studies met the inclusion criteria. Internal consistency was reported between 0.64 and 0.78 (Cronbach's alpha). Inter‐rater reliability was high, reported as ranging from 0.946 to 0.964 (intra‐class correlations) or 0.86 to 0.949 (Pearson's correlation). Most validity studies tested predictive validity with wide variances reported.

**Conclusion:**

The Braden Scale is reliable for assessing the risk of PI in acute care, but the validity of the scale is variable. Further research investigating validity beyond predictive validity is required.

**Relevance to Clinical Practice:**

Nurses working in acute care can use the Braden Scale with confidence of scale reliability. However, validity is variable and warrants a cautious approach. The true value resides in the capacity to trigger recognition of pressure injury risk.

**Trial Registration:** The protocol was registered a priori with the International Prospective Register of Systematic Reviews PROSPERO ref: CRD42023407545

**Summary**

- Pressure injury risk assessment is known to be a crucial step providing the base for implementing appropriate preventative interventions. The Braden Scale is the most widely used risk assessment scale in the acute care setting, yet validity and reliability is variable.
- This systematic review provides a consolidated understanding about the reliability and validity of the Braden Scale when used in the acute care setting.
- The Braden Scale is evidenced as a reasonably reliable tool when used in the acute care setting for pressure injury risk assessment. Reported validity of the Braden scale varies, with validity testing primarily focused on predictive validity.

## 1 Introduction

Pressure injuries (PI) are localised wounds to skin and underlying tissue which can decrease quality of life (Burston et al. 2022), prolong length of stay (Graves et al. 2023), increase morbidity (Jackson et al. 2018) and increase mortality (Song et al. 2019). PI are preventable, yet despite some decrease in incidence with recent advances in prevention strategies, rates remain relatively high (Fulbrook et al. 2023). For example, in one ICU‐specific study, the cumulative incidence of PI has been reported between 3.0% and 34.4% (Chaboyer et al. 2018). Recently, incidence across a sample of Finnish hospitals was reported at 10.0% (Tervo‐Heikkinen et al. 2022). The prevalence of PI in Australian public hospitals is estimated at 7.9% (Rodgers et al. 2021) with an associated estimated total loss of $9.11 billion on reparation costs (Nghiem et al. 2022).

PI results from the combinations of shear, friction, and pressure, due to contact with either a medical device or support service (Gefen et al. 2022). Their development can be attributed to both iatrogenic (Cox et al. 2020) and intrinsic risk factors (Al Aboud and Manna 2023), and PI prevalence can be reduced through increased transparency of nursing processes (Sim et al. 2024). The process of risk assessment is a key part of this nursing process, preceding the implementation of evidence‐based preventative strategies (Cox et al. 2020; Hultin et al. 2022). To support the use of appropriate prevention strategies, the use of a structured risk assessment supported by clinical judgement is recommended (EPUAP, NPIAP, and PPPIA 2019). Commonly, structured risk assessment is formatted as a list of prominent risk factors that predispose PI development, resulting in a score that reflects the patient's risk level (Moore and Patton 2019).

Current PI risk assessment tools vary considerably in their psychometric properties (reliability and validity). Psychometric properties demonstrate the extent to which a clinical tool measures what it is intended to measure, how stable it is over time and between users, and its ability to detect changes in conditions. However, in the case of PI risk assessment, there is some controversy regarding which risk factors (measured by PI risk assessment tools) have the greatest effect on PI development, with indications that they vary according to the patient cohort (Alderden et al. 2017; Moore and Patton 2019). Consequently, the reliability and validity of these risk assessment instruments are cohort dependent.

Of all the instruments available, the Braden Scale is used most widely across a broad range of clinical settings (Lovegrove et al. 2024), and is the most investigated in terms of its psychometric properties (European Pressure Ulcer Advisory Panel [EPUAP], National Pressure Injury Advisory Panel [NPIAP], Pan Pacific Pressure Injury Alliance [PPPIA] 2019). However, the Braden Scale was originally designed for use in long‐term care settings, with testing during its development attesting to the scale's content and construct validity, with two studies reporting its predictive validity and three studies reporting its reliability (Bergstrom, Braden, et al. 1987). It comprises six sub‐scales: activity, friction and shear, mobility, moisture, nutrition, and sensory perception (Bergstrom, Braden, et al. 1987). Each sub‐scale is scored from 4 to 1 except friction and shear, which is scored from 3 to 1 (Bergstrom, Demuth, and Braden (1987)), with lower scores indicating greater risk. The Braden Scale has been tested in diverse clinical settings in multiple countries with conflicting results of its validity and reliability (Chen et al. 2017; Huang et al. 2021; Šateková et al. 2017; Wei et al. 2020). Additionally, there is a paucity of quality evidence attesting to the efficacy of structured PI risk assessment tools when compared to nurses' use of clinical judgement in place of structured risk assessment; consequently, there is limited clarity as to which method of risk assessment is most effective to assess PI risk (Lovegrove et al. 2023; Moore and Patton 2019). Furthermore, although there is widespread use of the Braden Scale in acute clinical settings, to date there has been no rigorous investigation of the tool's psychometric properties for this patient cohort.

## 2 Aim

The aim of this systematic review was to analyse the existing evidence on the psychometric properties of the Braden scale when used within the acute care setting. The objective of the review was to provide clinicians with a synthesised understanding of the quality of the Braden scale as a commonly used instrument for assessing risk of PI in the acute care setting.

## 3 Methods

### 3.1 Design

A protocol for the systematic review was registered a priori with the International Prospective Register of Systematic Reviews PROSPERO (ref: CRD42023407545). This systematic review comprises the second stage of this registered protocol, with the first stage reporting on the intensive care setting (Mehicic et al. 2024). No funding was used to support the review. The PRISMA‐COSMIN Outcome Measurement Instruments guideline (Elsman et al. 2024) is an independent instrument based upon the Preferred Reporting Items for Systematic Reviews and Meta‐Analyses (PRISMA) guideline (Page et al. 2021) and has been used for reporting this review. The PRISMA‐COSMIN OMI is designed specifically for reporting studies of outcome measurement instruments evaluating at least one measurement property (Elsman et al. 2024).

### 3.2 Information Sources and Search Strategy

The Population (adult patients admitted to acute hospital settings), Intervention (Braden Scale risk assessment), Comparison (nil), Outcome (psychometric properties) (PICO) framework was used to develop the search strategy. MESH terms and keywords based upon PICO were developed, and Boolean operators (AND, OR) used to combine search terms. The initial search was undertaken in June 2023 as part of a broader systematic review, using Cumulative Index to Nursing and Allied Health Literature (CINAHL) Complete, Ovid Excerpta Medica database (EMBASE), EBSCO Medical Literature Analysis and Retrieval System Online (MEDLINE) Complete, Scopus and Web of Science (see Search Strategy example, Supporting Information S1). An updated search using the same search terms and databases was undertaken in February 2024 to capture articles published during 2023. All studies identified for inclusion were cross‐referenced with studies included in a larger systematic review by the authors (unpublished) of all risk assessment scales. Additionally, the screening of reference lists of systematic reviews found in the search was undertaken to identify potential articles for inclusion.

### 3.3 Eligibility Criteria

Studies included in the review had tested at least one psychometric property of the Braden Scale in a sample of adult patients (aged ≥ 18 years). For studies assessing reliability, a sample of nurse‐raters must have been included. The study was limited to acute care (non‐ICU) settings, or studies that included and reported an acute care setting as a sub‐set. Only peer‐reviewed primary quantitative or mixed‐methods research studies were included, with studies conducted in ICU, grey literature, editorials, conference papers, non‐peer reviewed articles from internet websites, and qualitative studies excluded. Any studies reporting data used to develop the original instrument were excluded, as inclusion of these could lead to overly optimistic results (Streiner and Kottner 2014). Studies were limited to those published in English, and no date limits were set.

### 3.4 Search Outcome and Selection Process

References identified through database searches were exported into EndNote 20 for duplicate removal, and then transferred into Covidence for screening, selection, and data extraction. A team of three primary reviewers undertook title and abstract screening, with each reference screened independently by two of these three reviewers. A fourth reviewer arbitrated conflicts. This same process was then used for full‐text screening of articles. Studies meeting the inclusion criteria progressed to quality appraisal and data extraction.

### 3.5 Quality Appraisal

Risk of bias was assessed manually, using the Consensus‐based Standards for the selection of health Measurement Instruments (COSMIN) checklist (Mokkink et al. 2018). This checklist is used for assessment of the methodological quality of each study included in a review, supporting the validity and reliability of the review findings. Independent assessment was conducted by two of three primary reviewers for each article, with a fourth reviewer arbitrating discrepancies. Results of risk of bias assessment were not used to exclude any studies from abstraction and synthesis.

### 3.6 Data Abstraction and Synthesis

A customised data extraction template was devised for this review. Two of the three reviewers independently extracted data from each article, with the following information collected: general information, study design and type of testing (reliability and/or reliability), participant population and setting, methodological approach, and main results. The fourth reviewer arbitrated conflicts to reach consensus. Due to the heterogeneity of the included studies, data were consolidated, presented in a tabulated format, and synthesised narratively.

## 4 Results

### 4.1 Study Selection

**FIGURE 1:**
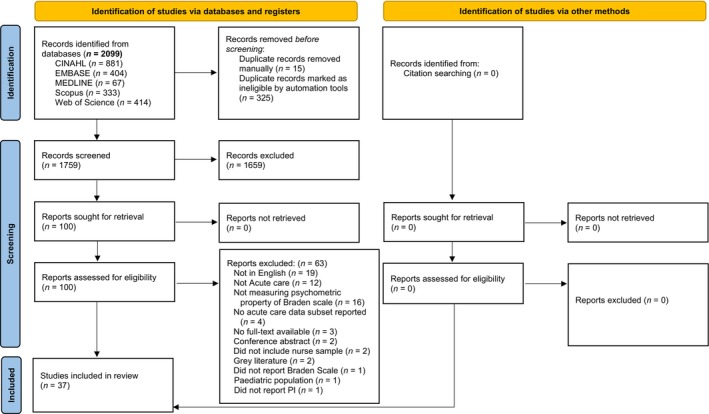
PRISMA flow diagram. *Source:* Page MJ, McKenzie JE, Bossuyt PM, Boutron I, Hoffmann TC, Mulrow CD, et al. The PRISMA 2020 statement: An updated guideline for reporting systematic reviews. BMJ 2021;372:N71. doi: 10.1136/bmj.n71. For more information, visit: http://www.prisma‐statement.org/. [Colour figure can be viewed at wileyonlinelibrary.com]

In total, 2099 articles were identified from database searching, and none from citation searching. After removing duplicates, titles and abstracts of 1759 articles were screened. A further 1659 studies were excluded, leaving 100 articles for full‐text screening. Following full‐text screening, 37 studies were deemed to meet all inclusion criteria and were included in this review. The study selection process and reasons for exclusion are detailed in Figure 1.

### 4.2 Risk of Bias

Two of the three primary reviewers independently assessed each study for risk of bias, with the fourth reviewer arbitrating. Results of studies testing reliability were mixed, ranging from ‘very good through to inadequate’ according to the COSMIN risk of bias checklist. Similarly, validity studies were judged widely ranging from ‘very good’ to ‘inadequate’. Table 1 details the results of risk of bias assessments.

**TABLE 1:** Risk of bias assessment.

Internal consistency						
Study	Does the scale consist of effect indicators, that is, is it based on a reflective model?	Was an internal consistency statistic calculated for each unidimensional scale or subscale separately?	For continuous scores: Was Cronbach's alpha or omega calculated?	For dichotomous scores: Was Cronbach's alpha or KR‐ 20 calculated?	For IRT‐based scores: Was standard error of the theta (SE (θ)) or reliability coefficient of estimated latent trait value (index of (subject or item) separation) calculated?	Were there any other important flaws in the design or statistical methods of the study?
Halfens et al. 2000	I	VG	VG—Cronbach's alpha	Cronbach's alpha	NA	NA
Stevens et al. 2024	VG	VG	VG—Cronbach's alpha	NA	NA	< 30 patients

Interrater reliability								
Study	Were patients stable in the interim period on the construct to be measured?	Was the time interval appropriate?	Were the test conditions similar for the measurements? For example, type of administration, environment, instructions	For continuous scores: Was an intraclass correlation coefficient (ICC) calculated?	For dichotomous/nominal/ordinal scores: Was kappa calculated?	For ordinal scores: Was a weighted kappa calculated?	For ordinal scores: Was the weighting scheme described? For example, linear, quadratic	Were there any other important flaws in the design or statistical methods of the study?
Barnes and Payton 1993	D	D	D	I	NA	NA	NA	NA
Choi et al. 2014	VG	VG	VG	NA	I	I	NA	I—Description of PROM I—Inclusion/Exclusion criteria
Halfens et al. 2000	D	D	A	I	VG	D	NA	n/a
Harrison et al. 1996	VG	VG	VG	I	NA	NA	NA	I—Inclusion/Exclusion criteria
Magnan & Makleburst 2008	D	D	A	I	I	I	I	Calculated reliable versus unreliable only; expert and RN assessments made at different times; 17 cases excluded with reasons
Rogenski and Kurcgant 2012	A	VG	VG	VG	VG	D	NA	I—Inclusion/Exclusion criteria
Wang et al. 2015	D	D	VG	VG	n/a	D	D	D—Description of development process D—Origin of construct
Zhao et al. 2022	VG	VG	VG	I	VG	D	I	NA

Predictive validity			
Study	For continuous scores: Were correlations, or the area under the receiver operating curve calculated?	For dichotomous scores: Were sensitivity and specificity determined?	Were there any other important flaws in the design or statistical methods of the study?
Baldwin and Ziegler 1998	I	VG	D—Representativeness of sample
Balzer et al. 2007	NA	VG	I—Inclusion/exclusion criteria
Barnes and Payton 1993	I	NA	NA
Bergstrom, Braden, et al. 1987	NA	VG	NA
Bergstrom et al. 1998	I	VG	NA
Capobianco and McDonald 1996	NA	VG	I—Inclusion/exclusion criteria
Chan et al. 2009	VG	VG	NA
Chen et al. 2023	VG	VG	NA
Dweekat et al. 2022	VG	VG	NA
Fu et al. 2023	VG	VG	NA
González‐Ruiz et al. 2014	VG	VG	I—Inclusion/exclusion criteria ROC analysis was undertaken but AUC was not reported
Gurkan et al. 2022	VG	VG	NA
Halfens et al. 2000	I	VG	NA
Harrison et al. 1996	I	VG	I—Inclusion/exclusion criteria
Jalali and Rezaie 2005	I	VG	NA
Jin et al. 2015	VG	VG	NA
Kallman & Lindgren, 2014	VG	VG	NA
Kumari et al. 2015	I	VG	D—origin of construct description ROC was analysed but AUC was not reported
Kwong et al. 2005	I	VG	NA
Lim et al. 2019	I	VG	NA
Lyder et al. 1998	I	VG	NA
Lyder et al. 1999	I	VG	NA
Miller et al. 2016	I	I	NA
Pouzols et al. 2023	VG	VG	NA
Saleh et al. 2009	VG	NA	I—Description of construct D—Description of development process D—Origin of construct I—Description of PROM D—Description of existing evidence
Salvadalena et al. 1992	I	VG	NA
Serpa and Santos 2014	NA	I	NA
VandenBosch et al. 1996	I	VG	NA
Watts et al. 1998	I	NA	NA

Convergent validity				
Study	Is it clear what the comparator instrument(s) measure(s)?	Were the measurement properties of the comparator instrument(s) sufficient?	Was the statistical method appropriate for the hypotheses to be tested?	Were there any other important flaws in the design or statistical methods of the study?
Tannen et al. 2010	VG	A	A	I—Inclusion/exclusion criteria

Criterion validity			
Study	For continuous scores: Were correlations, or the area under the receiver operating curve calculated?	For dichotomous scores: Were sensitivity and specificity determined?	Were there any other important flaws in the design or statistical methods of the study?
Gould et al. 2002	I	I	I—Inclusion/exclusion criteria
Harrison et al. 1996	I	VG	I—Inclusion/exclusion criteria

Diagnostic accuracy			
Study	For continuous scores: Were correlations, or the area under the receiver operating curve calculated?	For dichotomous scores: Were sensitivity and specificity determined?	Were there any other important flaws in the design or statistical methods of the study?
Tannen et al. 2010	VG	VG	I—Inclusion/exclusion criteria

Abbreviations: A, adequate; D, doubtful; I, inadequate; NA, not applicable; VG, very good.

^a^ COSMIN Risk of Bias checklist adapted from (Mokkink et al. 2018).

### 4.3 Characteristics of Included Studies

Studies were conducted in 16 countries and included patient sample sizes ranging from *one* (case study) to 24,277 (retrospective chart audit) and, when reported, nurse‐rater sample size ranged from *one* to 236; however, in about one‐third of studies (*n* = 13) the sample size was unclear or not reported. Most studies (*n* = 32) were prospective in nature. Predictive validity was the most common psychometric property tested (*n* = 29), followed by inter‐rater reliability (*n* = 7). Many studies (*n* = 14) used at least one comparator risk assessment tool. Characteristics of the included studies are summarised in Table 2.

**TABLE 2:** Characteristics of included studies.

Author, year	Country	Design	Aim	Psychometric test(s) reported	Setting and patient sample size (*n*)	Nurse‐rater(s) and sample size (*n*) or data source	Comparator risk assessment tool
Baldwin and Ziegler 1998	USA	Prospective	To identify factors associated with the incidence, number, stage, and location of pressure ulcers and to test the predictive validity of the Braden scale in a sample of previously healthy, critically injured trauma patients	Predictive validity	Hospital *n =* 36	Registered nurses (*n* = 5)	NA
Balzer et al. 2007	Germany	Prospective	To compare the sensitivity and specificity of the Norton, Waterlow, and Braden Scales in identifying patients at pressure sore risk	Predictive validity	Three hospitals *n* = 754	Ward nurses, other nurses, special data collectors *n* = NS	Norton, Waterlow
Barnes and Payton 1993	USA	Prospective	To identify factors associated with pressure‐sore formation	Predictive validity and Inter‐rater reliability	Tertiary acute care facility *n* = 361	Registered nurses *n* = 6	NA
Bergstrom, Demuth, and Braden (1987)	USA	Prospective	To determine sensitivity and specificity of the scale	Predictive validity	Teaching hospital V‐1 *n* = 99 V‐2 *n* = 100	V‐1 *n* = unclear V‐2 *n* = unclear	NA
Bergstrom et al. 1998	USA	Prospective	To evaluate the predictive validity of the Braden Scale in a variety of settings (tertiary care hospitals, Veterans Administration Medical Centers [VAMCs], and skilled nursing facilities [SNFs])	Predictive validity	Tertiary hospital, Medical center *n* = 843	Nurse 1—assessed level of risk, nurse 2 assessed skin condition	NA
Capobianco and McDonald 1996	USA	Prospective	To examine the predictive validity of the Braden scale using a cut‐off score of 18 for medical/surgical patients	Predictive validity	Teaching hospital *n* = 50	Registered nurse, Research nurse *n* = 2	NA
Chan et al. 2009	China (Hong Kong)	Prospective	To assess and compare the predictive validity of the modified Braden and Braden scales and to identify which of the modified Braden subscales are predictive in assessing pressure ulcer risk among orthopaedic patients in an acute care setting	Predictive validity	Hospital *n* = 197	Registered nurse *n* = 1	Modified Braden
Chen et al. 2023	China	Prospective	The overall aim was to provide a reference for screening patients at high risk of IAPI	Predictive validity	Hospital *n* = 100	Research nurses *n* = NS	Munro, Scott Triggers, CORN Intraoperative
Choi et al. 2014	USA	Prospective	To better understand how vague descriptions of patient characteristics may impact the practice of assessing pressure‐ulcer risk	Inter‐rater reliability	Two academic medical centres *n* = 102	*n* = 102	NA
Dweekat et al. 2022	USA	Retrospective	To develop an integrated system of Braden and machine learning to predict HAPI and assist with resource allocation for early interventions	Predictive validity	Hospital *n* = 15, 889	*n* = NS	NA
Fu et al. 2023	China	Prospective	To analyse the prevalence characteristics of PI in older inpatients and the predictive validity of the Braden scale, in order to provide evidence for a reasonable and effective clinical application of the Braden scale among older inpatients.	Predictive validity	Tertiary hospital *n* = 13,064	Clinical nurses *n* = NS	NA
González‐Ruiz et al. 2014	Spain	Prospective	The aim of this study was to validate the Braden Scale and the Norton Scale modified by INSALUD (Norton‐MI) in an acute care hospital, as well as to determine the most suitable CPs to identify PrU risk in patients	Predictive validity	University hospital *n* = 1001	Trained nurse *n* = 1	Norton modified
Gould et al. 2002	England	Prospective	The aim of this study was to examine the validity of the three most commonly used RAS's compared to nurses' own clinical judgement	Criterion validity	Mixed clinical settings *n* = 4 case studies	*n* = 236	Norton, Waterlow
Gurkan et al. 2022	Turkey	Prospective	This study aims to determine the predictive power of the Norton, Braden and Waterlow scales in determining risk of pressure injury (PI) in surgical patients	Predictive validity	Surgical clinic *n* = 250	*n* = NS	Norton, Waterlow
Halfens et al. 2000	Netherlands	Prospective	This study investigated whether adding new risk factors can enhance the sensitivity and specificity of the Braden scale	Predictive validity, Internal consistency, Inter‐rater reliability	Three hospitals *n* = 320	Ward nurses *n* = 2 per ward	NA
Harrison et al. 1996	Canada	Prospective	Through a prospective study, the prevalence of pressure ulcers was determined, and pressure ulcer incidence was tracked to evaluate the accuracy of the Braden Scale for risk assessment	Criterion validity, Inter‐rater reliability	University teaching hospital *n* = 738	*n* = 23	NA
Jalali and Rezaie 2005	Iran	Prospective	To compare the predictive validity of four pressure ulcer risk assessment tools	Predictive validity	Three educational hospitals *n* = 230	*n* = 4 research nurses	Gosnell, Norton, Waterlow
Jin et al. 2015	Republic of Korea	Retrospective	The purpose of this study was to evaluate the predictive validity of the Braden scale	Predictive validity	University Hospital *n* = 5932 (*n* = 4938 non‐ICU)	*n* = NS	NA
Källman and Lindgren 2014	Sweden	Prospective	The aims of this study were to examine and compare the predictive validity of four risk assessment scales used for the prediction of pressure ulcer (PrU) development and to identify risk factors	Predictive validity	Hospital *n* = 346	*n* = 2 (different for each assessment)	Norton, Modified Norton, Risk Assessment Pressure Sore (RAPS)
Kumari et al. 2015	India	Prospective	The aims of this paper were to compare the predictive validity of three pressure ulcer (PU) risk scales—the Norton scale, the Braden scale, and the Waterlow scale—and to choose the most appropriate calculator for predicting PU risk in surgical wards of India	Predictive validity	Tertiary care hospitals *n* = 100	*n* = 3	Norton, Waterlow
Kwong et al. 2005	China	Prospective	The aim of this study was to develop a modified Braden scale, to evaluate its predictive validity, and to identify a more valid pressure ulcer risk calculator for application in acute care hospitals in Mainland China among the modified Braden, Braden, and Norton scales	Predictive validity	Two acute care hospitals *n* = 429	*n* = 3 (different for each assessment)	Norton, Modified Braden
Lim et al. 2019	Singapore	Retrospective	The aim of this study was to compare the pressure injury risk predictability between the individual Braden subscales and the total Braden scale in adult inpatients in Singapore	Predictive validity	Public university hospital *n* = 199 (medical records)	*n* = NS	NA
Lyder et al. 1998	USA	Prospective	The purpose of this pilot study was to examine the Braden Scale for Predicting Pressure Sore Risk and determine which variables predict pressure ulcer risk in Black and Latino/Hispanic elders	Predictive validity	Medical centre (medical/surgical units) *n* = 36	*n* = 3 trained, 1 assessed Braden	NA
Lyder et al. 1999	USA	Prospective	The purpose of this study was to examine the predictive validity of the Braden scale in predicting pressure sore risk and to determine the physiological and non‐physiological variables associated with the prediction of pressure ulcers in Black and Latino/Hispanic elders	Predictive validity	Tertiary teaching hospital *n* = 74	*n* = 3 trained, 1 assessed Braden	NA
Magnan and Maklebust 2008	USA	Prospective	To evaluate the effect of Web‐based Braden Scale training on the reliability and precision of pressure ulcer risk assessments made by registered nurses (RN) working in acute care settings	Inter‐rater reliability	Three medical centres *n* = 102	Expert raters *n* = NS Registered nurses *n* = NS	Expert raters completed 102 assessments. Registered nurses completed 398 assessments
Miller et al. 2016	USA	Retrospective	The aim of this retrospective study was to determine the extent to which Braden Scale scores and other nutrition screening parameters (body mass index, poor intake, and weight loss) predict PU development in general and heel and sacral ulcers specifically	Predictive validity	Medical center (Level 1 trauma) *n* = 230	*n* = NS	NA
Pouzols et al. 2023	Switzerland	Retrospective	To develop, deploy, and validate in an operational clinical setting a new artificial intelligence–based and time‐aware predictive model for the early detection of patients at risk of HAPI that is tailored to the inpatient population	Predictive validity	University hospital *n* = 24,277	*n* = NS	Random forest (RF) & long short‐term memory (LTSM) predictive computer models
Rogenski and Kurcgant 2012	Brazil	Prospective	To verify pressure ulcer prevalence in hospital units of adult patients in the University Hospital of the University of São Paulo (HU‐USP), and to verify interrater reliability in risk assessment, using the Braden Scale	Inter‐rater reliability	University Hospital *n* = 87	*n* = 6 research nurses	NA
Saleh et al. 2009	Jordan	Prospective	To determine whether use of a risk assessment scale reduces nosocomial pressure ulcers	Predictive validity	Military hospital *n* = 521 total *n* = 265 pre‐test *n* = 256 post‐test	*n* = 3	NA
Salvadalena et al. 1992	USA	Prospective	To evaluate the Braden scale for its efficacy in their acute care setting	Predictive validity	Community hospital *n* = 99	*n* = 5	NA
Serpa and Santos 2014	Brazil	Prospective	The purpose of this study was to analyse the validity of the nutrition subscale from the Braden Scale for Predicting Pressure Sore Risk in hospitalised patients	Predictive validity	Two private hospitals *n* = 170	*n* = 1 researcher *n* = 6 collaborators (3 in each hospital)	NA
Stevens et al. 2024	USA	Prospective	The goal of this study was to test the ability of an educational intervention tailored to specific interventions based on the subscales of the Braden Scale to improve knowledge among nurses	Internal consistency	Teaching hospital *n* = 1 case study	*n* = 38	NA
Tannen et al. 2010	Germany	Prospective	Comparison of the diagnostic accuracy of two pressure ulcer risk assessment scales and one generic nursing assessment tool	Convergent validity, Diagnostic accuracy	Hospitals and nursing homes *n* = 1053	*n* = unclear	Waterlow, Care Dependency Scale
VandenBosch et al. 1996	USA	Prospective	To determine the cut‐off point at which patients would be at risk for developing a pressure ulcer	Predictive validity	Tertiary community teaching hospital *n* = 103	*n* = 8	NA
Wang et al. 2015	China	Prospective	The objective of this study was to evaluate inter‐rater reliability of Braden Scale, Norton Scale and Waterlow Scale for pressure ulcer risk assessment in clinical practice	Inter‐rater reliability	Hospital *n* = 23	*n* = 6	Norton, Waterlow
Watts et al. 1998	USA	Prospective	The purpose of this study was to determine the prevalence, location, degree, and predictors of skin breakdown in trauma patients, as well as the diagnosis groups most at risk	Predictive validity	Hospital *n* = 148	*n* = unclear	NA
Zhao et al. 2022	China	Prospective	To develop and evaluate a cartoon version of the Braden scale (CVBS)	Inter‐rater reliability	*n* = 239	*n* = 46 wound care specialists *n* = 114 bedside nurses	Braden Cartoon version

Abbreviations: NA, not applicable; NS, not stated.

Two studies reported internal consistency with Cronbach's alpha of 0.78 [original scale] and 0.76 [extended scale] (Halfens et al. 2000), and 0.64 (Stevens et al. 2024). Seven studies tested inter‐rater reliability (Barnes and Payton 1993; Halfens et al. 2000; Harrison et al. 1996; Magnan and Maklebust 2008; Rogenski and Kurcgant 2012; Wang et al. 2015; Zhao et al. 2022) using Pearson's correlation, intra‐class correlation, or kappa; however, none reported instrument measurement error (see Table 3). One study reported on the reliability of nurses' interpretations of scale descriptors but did not conduct statistical testing (Choi et al. 2014).

**TABLE 3:** Interrater reliability: Main results.

Author, year	Inter‐rater reliability	Measurement error
Barnes and Payton 1993	Pearson's product moment correlation *r* = 0.86	NR
Choi et al. 2014	NR (tested nurses' interpretations of Braden scale descriptors)	NR
Halfens et al. 2000	Kappa *r* = 0.86 Braden scale	NR
Harrison et al. 1996	Pearson's product moment correlation *r* = 0.87	NR
Magnan and Maklebust 2008	Pre‐test 62.1% reliable Post‐test 64.6% reliable [^a^ ^s^reliability determined with kappa ≥ 0.549 (*p* = 0.008)]	NR
Rogenski and Kurcgant 2012	Pearson correlation = 0.949, Intraclass correlation (ICC) = 0.946	NR
Wang et al. 2015	Intraclass correlation (ICC) = 0.964 (95% CI 0.827)	NR
Zhao et al. 2022	Kappa: Sensory 0.83 (0.77–0.81) Moisture 0.77 (0.69–0.84) Activity 0.80 (0.70–0.89) Mobility 0.80 (0.72–0.86) Nutrition 0.69 (0.60–0.76) Friction and shear 0.60 (0.51–0.69) Risk rating 0.69 (0.61–0.76)	NR “The lower the consistency of the subcategories, the greater the measurement error.” (p. 399)

^a^ CI = confidence interval, MDC = minimal detectable change, SEM = standard error of measurement, NR = not reported.

Predictive validity was tested in 29 studies (see Table 4), of which most (*n* = 26) tested predictive validity alone. Criterion validity was examined in two studies (Gould et al. 2002; Harrison et al. 1996), with convergent validity and diagnostic accuracy investigated in one study (Tannen et al. 2010). When reporting predictive validity of the Braden Scale, reported cut‐off scores ranged from 10 to 20, sensitivity from 16.2 to 100%, specificity from 0% to 100%, positive predictive value (PPV) from 1.2% to 100%, negative predictive value (NPV) from 0% to 100%, and the area under curve (AUC) from 54.9% to 90.5%.

**TABLE 4:** Predictive validity: Main results.

Author, year	Cut‐off score (reported)	Sensitivity %	Specificity %	PPV %	NPV %	AUC %	Comments
Baldwin and Ziegler 1998	10	91.0	96.0	NR	NR	NR	Best cut off score = 9 (YI 1.0)^a^ Sensitivity % = 100 Specificity % = 100
Balzer et al. 2007	19	81.0	72.0	NR	NR	NR	
Barnes and Payton 1993	16	73.0	91.0	NR	NR	NR	
Bergstrom, Braden, et al. 1987	16	100.0	90.0	NR	NR	NR	Validation study 1 Validation study 2
	16	100.0	64.0	NR	NR	NR	
Bergstrom et al. 1998	18	88.0	68.0	21.0	99.0	NR	Tertiary Care
	18	70.0	79.0	78	96	NR	VAMC
	18	81.0	73.0	50	92	NR	SNF
Capobianco and McDonald 1996	18	71.0	83.0	63.0	88.0	NR	Best cut off = 19 (YI 0.64)^a^ Sensitivity % = 86 Specificity % = 78
Chan et al. 2009	16	67.0	64.0	NR	NR	68.4 (95% CI 50.9–78.6)	Modified Braden best cut off = 19 (YI 0.51)^a^ Sensitivity % = 89.0 Specificity % = 62.0
Chen et al. 2023	16	16.2	93.7	60.0	65.6	63.0	Pre‐operative
	16	100	0	37.0	0.0	54.9	Intra‐operative
Dweekat et al. 2022	NR	60.6	58.1	NR	NR	59.4 (SD‐2.2)	Training set admission FPR % = 41.9 (SD‐0.7) Detection prevalence % = 42.8 (SD‐0.6)
	NR	66.9	59.0	NR	NR	62.3 (SD‐3.1)	Testing set admission FPR % = 41.1 (SD‐1.4) Detection prevalence % = 42.0 (SD‐ 0.4)
	NR	74.0	59.0	NR	NR	66.6 (SD‐1.0)	Training set before discharge FPR % = 41.0 (SD‐1.4) Detection prevalence % = 42.3 (SD‐1.4)
	NR	72.7	62.5	NR	NR	68.1 (SD‐3.0)	Testing set before discharge FPR % = 37.5 (SD‐1.1) Detection prevalence % = 38.8 (SD‐1.1)
Fu et al. 2023	19	84.6	86.4	1.2	99.9	90.5 (95% CI 84.4–96.5)	Sensory perception and moisture subscales had poor accuracy in predicting the PI risk of older hospitalised patients
González‐Ruiz et al. 2014	16	65.7	79.9	19.4	97.4	83.2 (95% CI 80.7–84.9)	Best cut off = 18 (YI 0.54)^a^ Sensitivity % = 87.5 Specificity % = 66.5
Gurkan et al. 2022	16	100	40.4	18.6	100	77.1	
Halfens et al. 2000	20	73.7	70.1	NR	NR	NR	Best cut‐off extended Braden = 23 (YI 0.43)^a^ Sensitivity % = 73.7 Specificity % = 69.4
Harrison et al. 1996	16	38.0	87.0	30.0	91.0	NR	Best cut‐off = 19 (YI 0.33)^a^ Sensitivity % = 67.0 Specificity % = 66.0
Jalali and Rezaie 2005	18	53.0	100	100	58.0	NR	
Jin et al. 2015	19 (YI = 0.67)	75.0	92.0	46.0	97.0	NR	First Braden score after admission
	19 (YI = 0.78)	88.0	90.0	45.0	99.0	NR	Last Braden score after admission
	19 (YI = 0.75)	90.0	85.0	37.0	99.0	NR	Lowest Braden score from admission to discharge *or* pressure injury development
Källman and Lindgren 2014	18	74.5	73.7	35.0	93.8	78.7	Best cut off = 19 (YI 0.49)^a^ Sensitivity % = 81.8 Specificity % = 67.1
Kumari et al. 2015	16	87.0	93.5	44.4	96.0	NR	Best cut off = 17 (YI 0.90)^a^ Sensitivity % = 100 Specificity % = 89.6
Kwong et al. 2005	14	89.0	72.0	5.0	100	NR	
Lim et al. 2019	17	75.0	68.0	68 (true positive)	74 (true negative)	NR	
Lyder et al. 1998	16	35.0	100	100	71.0	NR	
Lyder et al. 1999	16	90.0	14.0	60.0	50.0	NR	Latino/Hispanics ≤ 74 years
	16	77.0	50.0	77.0	50.0	NR	Blacks ≤ 74 years
	18	81.0	100	100	60.0	NR	Blacks ≥ 75 years
Miller et al. 2016	NR	NR	NR	NR	NR	NR	Week 1 HAPU: OR 0.64 (95% CI 0.46–0.89), *p* = 0.009 Week 2 HAPU: OR 0.71 (95% CI 0.47–1.08), *p* = 0.0113 Braden score day 7, OR 0.37 (95% CI = 0.02–0.69), *p* = 0.002
Pouzols et al. 2023	18	88.0	61.0	NR	NR	72.0	Accuracy = 0.61
Saleh et al. 2009	18	NR	NR	NR	NR	62.7	Pre‐test
	18	NR	NR	NR	NR	65.8	Post‐test
Salvadalena et al. 1992	19	80.0	43.0	26.0	89.0	NR	Best cut off = 19 (YI 0.23)^a^ Sensitivity % = 80.0 Specificity % = 43.0
Serpa and Santos 2014	NR	NR	NR	NR	NR	NR	The most statistically significant nutritional variables for PI development were the total Braden Scale score (moderate risk OR = 2.552 (95% CI 1.556–3.339, *p* < 0.001); high risk OR = 2.776 (95% CI 1.138–3.574, *p* < 0.001), Braden nutrition score, Very poor OR = 2.937 (95% CI 2.189–3.942, *p* < 0.001)
VandenBosch et al. 1996	17	59.0	59.0	NR	NR	NR	
Watts et al. 1998	18	100	42.0	NR	NR	NR	Best cut off = 14 (YI 0.64)^a^ Sensitivity % = 90.0 Specificity % = 74.0

Abbreviations: AUC, area under the curve; CI, confidence interval; LR, likelihood ratio; NPV, negative predictive value; NR, not reported; OR, odds ratio; PI, pressure injury; PPV, positive predictive value; YI, Youden Index.

^a^ Denotes the best cut‐off score—calculated *post hoc* using Youden's Index and included when different from reported best cut‐off score or not reported.

## 5 Discussion

The aim of this study was to analyse the psychometric properties of the Braden Scale when used to assess PI risk in adults within an acute care setting. The objective was to provide clinicians with a synthesised understanding of the quality of the Braden Scale for assessing risk of PI in the acute care setting.

### 5.1 Validity

The validity of a scale refers to the degree to which a scale measures that which it is purported to measure. Our review identified three types of validity testing for the Braden Scale: predictive, convergent and criterion validity. Predictive validity is a measure of how well the scale predicts a future outcome (occurrence of PI). Convergent validity is determined through comparison with other scales claimed to measure the construct (risk of PI). Criterion validity is considered with the accuracy of a scale to measure a future outcome (PI). An important finding of this review is that most studies reported on predictive validity when testing the psychometric properties of the Braden Scale in the acute care setting.

Predictive validity is important when assessing risk; however, within the context of PI risk assessment the value is debated. The value of risk assessment scales in PI prevention resides mainly in the risk identification aspect of the scale, which is used to justify clinical interventions to reduce the risk of PI occurrence (European Pressure Ulcer Advisory Panel [EPUAP], National Pressure Injury Advisory Panel [NPIAP], Pan Pacific Pressure Injury Alliance [PPPIA] 2019; Fulbrook and Anderson 2016). The Braden Scale is not a diagnostic tool but one of risk identification to support evidence‐based interventions, and as such, the psychometric validity and ultimate usefulness of the scale must be viewed in this light.

The cut‐off score of an instrument is important in supporting the correct use of the score, in the context of Braden Scale the cut‐off score is used to determine patients ‘at risk’ of pressure injury. Identification of those ‘at risk’ is crucial for implementation of the preventative strategies used to reduce risk, and as such an accurate cut‐off score is clinically important to patient outcomes. Initial validation work by Bergstrom, Braden, et al. (1987) recommended a cut‐off score of ≤ 16 with their follow‐up study recommending ≤ 18 (Bergstrom et al. 1998). Across this review a wide range of best cut‐off scores were reported, ranging from as low as 10 to as high as 20. This range indicates a significant variation in the recommended point at which a patient is deemed ‘at risk’, and therefore compromises the implementation of timely preventative interventions.

The ‘optimal’ cut‐off score is that which identifies the most correct and the least incorrect cases (Perkins and Schisterman 2006). Youden's Index (YI) calculations combines both the sensitivity and specificity, to ascertain the best balance between the two constructs and hence the most effective cut‐off point (Liu 2012; Fluss et al. 2005). In our review we identified only one of the 29 studies reporting use of this method (Jin et al. 2015). Therefore we undertook *post hoc* calculation of YI for all included predictive validity studies, finding that the reported best cut‐off score for 10 studies was contradictory to that which we identified using the YI calculation (Baldwin and Ziegler 1998; Capobianco and McDonald 1996; Chan et al. 2009; González‐Ruiz et al. 2014; Halfens et al. 2000; Harrison et al. 1996; Källman and Lindgren 2014; Kumari et al. 2015; Salvadalena et al. 1992; Watts et al. 1998). The differences between reported and calculated cut off scores ranged between one and four points (see Table 4). Additionally, when reported, wide variance in the sensitivity (16.2% to 100%), specificity (0% to 100%), positive predictive value (1.2% to 100%), and negative predictive value (0% to 100%) of the Braden Scale was identified (Table 4). This variability is not unique and has been reported in diverse settings such as ICU (Mehicic et al. 2024), long‐term care (Wilchesky and Lungu 2015), and critical care (Cox 2012).

### 5.2 Reliability

Reliability refers to the capacity of an assessment scale to measure a construct consistently, which for the Braden Scale is risk of PI. Inter‐rater reliability is one measure of reliability and measures the consistency of scale outcomes when used for the same assessment by different raters. PI risk assessment has long been a component of daily nursing practice and, as such, reliability of assessment scale use should be high. All eight studies reporting reliability identified in this review tested inter‐rater reliability.

Seven of the eight studies reported statistical calculations for reliability, with Choi et al. (2014) instead testing nurses' interpretations of five subjective Braden Scale descriptors (over most of body, often but not always, walks occasionally, very short distances, occasionally slides down). Reliability in interpreting these subjective descriptors varied depending on clinical context and years of experience, with mean percentage agreement between expert nurses and ward nurses ranging from 33.3% to 50%. Overall, Choi et al. (2014) concluded that significant variation exists when nurses interpreted these subjective descriptors. The subjectivity of Braden Scale items is recognised (Miller et al. 2020) and ultimately this may undermine the reliability of the instrument as an accurate risk assessment tool.

Intraclass correlation (ICC) is the most appropriate mechanism for assessing the reliability of repeated measures on a scale (Streiner and Kottner 2014; de Vet et al. 2006); however, it was only reported in two of the eight studies. Of these, Rogenski and Kurcgant (2012) reported an ICC of 0.95 (CI not reported) and Wang et al. (2015) an ICC of 0.96 (95% CI 0.83), both indicating good reliability of the Braden Scale (Koo and Li 2016). Pearson's product moment correlation was reported in three studies respectively as *r* = 0.95 (Rogenski and Kurcgant 2012), *r* = 0.87 (Harrison et al. 1996), and *r* = 0.86 (Barnes and Payton 1993), indicating generally strong positive correlations. Kappa for the complete Braden Scale was reported in two studies. A kappa of ≥ 0.55 (*p* = 0.008) was reported by Magnan and Maklebust (2008) indicating moderate agreement, with Halfens et al. (2000) reporting a kappa = 0.86, indicating near perfect agreement. Zhao et al. (2022) reported kappa statistics for Braden sub‐scales only, with results ranging from 0.60 (friction and shear) to 0.83 (sensory), indicating moderate to substantial agreement at the sub‐scale level.

Standard error of measurement is an important psychometric indicator of instrument reliability, most specifically the precision of an instrument in measuring what it intends to measure. In this context, the precision of the Braden scale to measure PI risk factors is noted. Standard error of measurement was not calculated in any of the eight studies reporting reliability testing. Zhao et al. (2022), when reporting inter‐rater reliability of Braden sub‐scales, did briefly note the relationship between the consistency of sub‐scales and measurement error. This lack of testing for agreement impacts negatively, therefore, on the capacity to detect clinically important changes (Lovegrove et al. 2024).

The findings of this review present some important implications for clinical practice and future research. When used to assess the risk of PI for adult patients in the acute care setting, the Braden Scale demonstrates good reliability, indicating it as a consistent and stable measurement instrument when used by clinicians. The validity of the Braden Scale varies widely, indicating that the accuracy of assessing risk using this scale is inconsistent. Most of the studies investigating validity that we identified assessed the predictive validity of the Braden Scale, that is, the accuracy of the scale in predicting the occurrence of a pressure injury. However, the purpose of a risk assessment tool like the Braden Scale is not about predicting occurrence, but of risk identification to support evidence‐based interventions. Therefore, the value of this psychometric validity, specifically predictive validity, and the ultimate clinical usefulness of the scale must be viewed in this light. As PI risk assessments like the Braden Scale form the basis for interventions to prevent PI, clinicians should not become reliant upon the Braden Scale as the only important aspect of risk assessment and should use appropriate clinical judgement in combination with the instrument. Future research seeking to examine and improve the validity of the instrument within this setting is warranted.

### 5.3 Limitations

The findings of this study relate only to the psychometric properties of the Braden Scale when used in the acute care setting, and as such findings are not generalizable to other settings. Only studies reported in English were included; consequently, relevant findings from non‐English language studies may have been excluded.

## 6 Conclusion

Pressure injury risk assessment remains an important step in identifying the risk of PI for acute care patients, used to support the implementation of evidence‐based interventions. However, to implement appropriate interventions with the highest likelihood of clinical effectiveness, risk assessment must be reliable and must be conducted with a valid assessment scale. Findings from our study indicate the Braden Scale is generally reliable for assessing the risk of PI in acute care, yet the validity of the scale remains uncertain. Additional research investigating validity properties beyond predictive validity is needed to better explore and clarify the validity of this scale, for example, criterion validity studies that could further refine the subjective descriptors that exist within the scale.

## Author Contributions

A.B.: Conceptualisation, methodology, validation, formal analysis, investigation, writing – original draft, review and editing, visualisation, project administration. J.B.: Methodology, formal analysis, investigation, writing – review and editing. A.M.: Methodology, investigation, writing – review and editing. P.F.: Conceptualisation, methodology, validation, formal analysis, investigation, writing – review and editing, visualisation, project administration.

## Ethics Statement

The authors have nothing to report.

## Conflicts of Interest

The authors declare no conflicts of interest.

## Supporting information


Data S1.

Data S2.

## Data Availability

Data sharing is not applicable to this article as no new data were created or analyzed in this study.
