# Supplementary material for: The Psychometric Properties of the Braden Scale to Assess Pressure Injury Risk in Acute Care: A Systematic Review
Source: J Clin Nurs. 2025 Jun 16;34(10):4055–73. doi: 10.1111/jocn.17862 (PMC12409248; doi:10.1111/jocn.17862)
Supplement: Supplementary file 1 — Data S1. [file JOCN-34-4055-s002.docx]

**Supplementary File 1.** Search strategy Key Words

| **Population** | **AND** | **Intervention AND Comparison** | **AND** | **Outcome** |
| --- | --- | --- | --- | --- |
| (hospital* OR clinical* OR  "acute care" OR "secondary care" OR "tertiary care" OR  "health facilit*" OR inpatient* OR patient* or iatrogenic) |  | ((pressure OR bed OR decubitus OR "deep tissue") **AND** (injur* OR ulcer* OR sore* OR wound*))  **AND** (risk*)  **AND** (braden*) |  | (psychomet* OR reliab* OR valid* OR accur* OR agree* OR consist*) |
